# Incidence and Prevalence of Frontotemporal Dementia: A Systematic Review and Meta-Analysis

**DOI:** 10.1001/jamaneurol.2025.3307

**Published:** 2025-09-08

**Authors:** Daniele Urso, Stefano Giannoni-Luza, Carol Brayne, Nicolas Ray, Giancarlo Logroscino

**Affiliations:** 1 Center for Neurodegenerative Diseases and the Aging Brain, University of Bari ‘Aldo Moro,’ “Pia Fondazione Cardinale G. Panico,” Tricase, Lecce, Italy; 2 GeoHealth Group, Institute of Global Health, Faculty of Medicine, University of Geneva, Geneva, Switzerland; 3 Cambridge Public Health, University of Cambridge, Cambridge, United Kingdom; 4 Department of Translational Biomedicine and Neuroscience (DiBraiN), University of Bari ‘Aldo Moro,’ Bari, Italy

## Abstract

**Importance:**

Comprehensive incidence and prevalence rates of frontotemporal dementia are currently not available.

**Objective:**

To estimate the incidence and prevalence of frontotemporal dementia and its clinical variants in the overall population and age subgroups.

**Data Sources and Study Selection:**

We systematically searched PubMed, EMBASE, and Scopus between January 1, 1990, and October 22, 2024, for population-based studies estimating the incidence and/or prevalence of FTD.

**Data Extraction and Synthesis:**

Studies and data were screened and extracted independently by 2 investigators in accordance with PRISMA guidelines. Incident and prevalent cases together with the population at risk were pooled using random-effects meta-analysis. Differences in heterogeneity by FTD variants and populations at risk were estimated.

**Main Outcomes and Measures:**

Prevalent and incident cases as numerator were based on well-defined clinical criteria. Denominators were derived either from census population data or from author-defined populations at risk.

**Results:**

From 1854 screened articles, 32 eligible population-based studies were identified. Sixteen were on prevalence and 22 on incidence reporting FTD measures, including those with estimates for the whole population and for specific age subgroups. Pooled crude incidence for FTD was 2.28 (95% CI, 1.55-3.36) per 100 000 person-years and prevalence, 9.17 (95% CI, 3.59-23.42) per 100 000 people. The behavioral-variant FTD pooled crude incidence was 1.20 (95% CI, 0.67-2.16) per 100 000 person-years and prevalence, 9.74 (95% CI, 2.90-32.73) per 100 000 people. The primary progressive aphasia variant pooled crude incidence was 0.52 (95% CI, 0.35-0.79) per 100 000 person-years and prevalence, 3.67 (95% CI, 3.05-4.43). FTD incidence among individuals younger than 65 years was 1.84 (95% CI, 0.79-4.30) per 100 000 person-years and prevalence, 7.47 (95% CI, 4.13-13.49) per 100 000 people. The denominator based on census data showed less heterogeneity than the population at risk defined by the authors (*I^2^*: for incidence, 91.6% vs 97.6%, respectively, and for prevalence, 98.8% vs 99.2%, respectively).

**Conclusions and Relevance:**

In this systematic review and meta-analysis, estimates indicate that FTD is comparable in frequency to dementia with Lewy bodies and occurs at higher rates than progressive supranuclear palsy, corticobasal syndrome, and amyotrophic lateral sclerosis. These results provide a foundation for future research and public health strategy, especially for underrepresented populations, to better comprehend the global burden of FTD.

Our findings provide robust pooled estimates of the incidence and prevalence of FTD and its subtypes, offering a foundation for future research and public health planning.

## Key Points

**Question:**

What is the incidence and prevalence of frontotemporal dementia (FTD)?

**Findings:**

In this systematic review and meta-analysis of population-based studies with an analyzed population of approximately 31 million person-years for incidence and 6 million individuals for prevalence spread in more than 12 regions around the world, FTD pooled incidence was 2.28 (95% CI, 1.55-3.36) per 100 000 person-years, and pooled prevalence was 9.17 (95% CI, 3.59-23.42) per 100 000 people.

**Meaning:**

With these results providing a robust pooled estimate of FTD incidence and prevalence, future research should include underrepresented populations and other world regions to better comprehend the global burden of FTD.

## Teaser

This systematic review and meta-analysis uses data from population-based studies to pool incident and prevalent cases of frontotemporal dementia together with the population at risk using random-effects meta-analysis.

## Introduction

Frontotemporal dementia (FTD) encompasses a group of clinically and pathologically heterogeneous neurodegenerative disorders characterized by progressive decline in behavior and language.^1^ The behavioral variant of FTD (bvFTD) predominantly presents with alterations in personality, executive dysfunction, and social behavior,^2^ whereas primary progressive aphasias (PPA) are defined by selective impairments in language, with subtypes including the semantic variant (svPPA) and the nonfluent/agrammatic variant (nfPPA).^3^ These syndromes share a common pathological hallmark of degeneration primarily affecting the frontal and anterior temporal lobes.^4,5^ FTD is a leading cause of early-onset dementia,^6^ defined as symptom onset before the age of 65 years, and accounts for a substantial proportion of neurodegenerative dementias within this age group.^6^ However, as with other neurodegenerative conditions, the incidence of FTD appears to increase with advancing age.^7^

Over the past 3 decades, diagnostic frameworks such as those developed by the Lund and Manchester Groups,^8^ Neary et al,^9^ and Rascovsky et al^2^ have provided robust criteria to identify FTD and its subtypes in both research and clinical practice. Despite advances in diagnostic approaches, there remains a critical gap in our understanding of the global incidence and prevalence of FTD. Population-based studies offer essential insights into the societal and health care burden of FTD, guiding resource allocation and informing public health strategies.^10^ However, challenges in study design, heterogeneity of diagnostic criteria, and limited geographic representation have hindered the synthesis of reliable estimates.^10,11^

To date, no meta-analysis has systematically addressed the global burden of FTD by pooling incidence and prevalence data across diverse populations.^11^ This lack of comprehensive evidence underscores the need for robust estimates to inform clinical practice and health care planning. In this systematic review and meta-analysis, we aim to provide the pooled estimates of FTD incidence and prevalence, including its subtypes, and to explore variations by age, geographic region, and methodological approaches. By addressing this gap, we hope to contribute to a deeper understanding of the epidemiology of FTD and its implications for global health policy.

## Methods

This systematic review with meta-analysis was conducted in accordance with our published protocol (PROSPERO CRD42024600983) and followed the Cochrane handbook^12^ and Preferred Reporting Items for Systematic Reviews and Meta-Analyses (PRISMA) reporting guidelines.^13^

### Study Types and Eligibility Criteria

We included population-based studies that reported the incidence or prevalence of FTD^2^ in global populations, focusing on bvFTD and PPA,^3^ including its subtypes (svPPA, nfPPA). Data on FTD associated with amyotrophic lateral sclerosis (FTD-ALS) were included. However, we excluded studies reporting on logopenic PPA, as this subtype is typically associated with Alzheimer pathology rather than frontotemporal degeneration.^14^ Eligible studies used validated diagnostic criteria such as frameworks from the Lund and Manchester Groups,^8^ Neary et al,^9^ Gorno-Tempini et al,^3^ or Rascovsky et al,^2^ or reported FTD diagnoses using *Diagnostic and Statistical Manual of Mental Disorders* (*DSM*) or *International Classification of Diseases* (*ICD*) classifications. The studies included used either a registry-based design or a 2- or 3-phase prevalence survey design. Registry-based studies typically follow a reconstructed cohort approach, where case information is collected from a well-defined geographic region through a surveillance system.^10,15^ This system integrates clinical case ascertainment with administrative population data to identify a theoretical cohort at risk, allowing for incidence and prevalence estimates.^15^ Two-phase prevalence surveys, on the other hand, use an initial large-scale screening phase with inexpensive but sensitive tools, followed by a second phase in which a subsample undergoes a more detailed diagnostic assessment.^16^ These designs are particularly useful for studying rare disorders, optimizing resource allocation, and improving case ascertainment accuracy.^15,17^ Studies were excluded if they were editorials, opinion pieces, or conference abstracts without associated full articles or did not provide sufficient empirical data for calculating incidence or prevalence estimates. For prevalence, studies using door-to-door methodologies were excluded because of their limitations in capturing accurate data for rare diseases.

### Search Strategy and Study Selection

A comprehensive search was conducted across PubMed, Embase, and Scopus from January 1, 1990, to October 22, 2024. The search strategy included terms such as “frontotemporal dementia,” “FTD,” “incidence,” “prevalence,” “behavioral variant FTD,” and “primary progressive aphasia” (eTable 1 in Supplement 1). The screening and selection process was conducted using the systematic review manager Covidence (Veritas Health Innovation). Duplicates were removed, after which 2 authors (D.U. and S.G-L) independently screened titles and abstracts. Full-text reviews were then performed on the retained articles. Any discrepancies during the selection process were resolved by consensus with a third researcher (G.L.). Reasons for exclusion at the full-text stage are detailed in eTable 2 in Supplement 1. Additionally, reference lists of included studies were thoroughly reviewed to identify any potentially missed articles.

### Data Extraction and Preparation

Two reviewers (D.U. and S.G-L.) independently collected study data using a standardized form. Extracted data included study characteristics such as author, year, country, and geographically defined area; target period, sample size, and population setting; diagnostic criteria and study design; incidence and prevalence data; and the age range of the population at risk, including age- and sex-specific incidence and prevalence data where available. Population at risk was extracted as defined by the authors. However, when authors did not explicitly define the denominator as encompassing the entire population (of all ages), we additionally calculated the denominator using census population data in the relevant geography for the specified target period. For studies including incidence and prevalence data for PPA that incorporated the logopenic variant, we subtracted the logopenic PPA cases from the numerator to estimate incidence and prevalence attributable solely to FTD. Disagreements were resolved by discussion or adjudication by a third reviewer (G.L.). Authors of included studies were contacted for clarification or additional data if necessary.

### Risk-of-Bias Assessment

The quality of included studies was assessed independently by 2 reviewers (D.U., S.G-L.) using the Joanna Briggs Institute Critical Appraisal Checklist for prevalence data and the Newcastle-Ottawa Scale for incidence studies. Domains evaluated included representativeness of the population, measurement validity, and consistency of diagnostic criteria. Discrepancies were resolved by a third reviewer.

### Statistical Analysis and Data Synthesis

Incidence and prevalence estimates were synthesized using random-effects meta-analysis models to account for heterogeneity across studies. Pooled estimates were calculated and presented with 95% CIs. Heterogeneity was quantified using the *I^2^* statistic and assessed with Cochran *Q* test. The primary analysis included only studies that reported incidence and prevalence data for the entire population, as defined by census data. Additionally, we meta-analyzed studies on specific age groups (<65 or ≥65 years) using population at risk as defined by authors. Subgroup analyses were conducted to explore variations by FTD subtypes (eg, bvFTD, FTD-ALS, nfPPA, svPPA, undifferentiated PPA), age groups, and methodological approaches (eg, registries, nationwide studies). Sensitivity analyses included the use of denominators defined by the authors, cumulative meta-analyses, and influence analyses. Data analyses were performed using the metarate and metaprop functions for incidence and prevalence respectively from the meta package, R version 4.4.2 (R Foundation). Forest plots were generated to illustrate individual and pooled estimates.

### Comparative Analysis With Other Neurodegenerative Diseases

To contextualize our findings, we conducted a focused review of the literature to retrieve population-based estimates of incidence and prevalence for other major neurodegenerative diseases, including Alzheimer disease (AD), Parkinson disease (PD), dementia with Lewy bodies (DLB), progressive supranuclear palsy (PSP), corticobasal syndrome (CBS), and ALS. When meta-analyses were not available, we included the most recent and methodologically robust single studies. Full details and sources are presented in eTables 3 and 4 in Supplement 1.

## Results

### Study Selection

**Figure 1.**
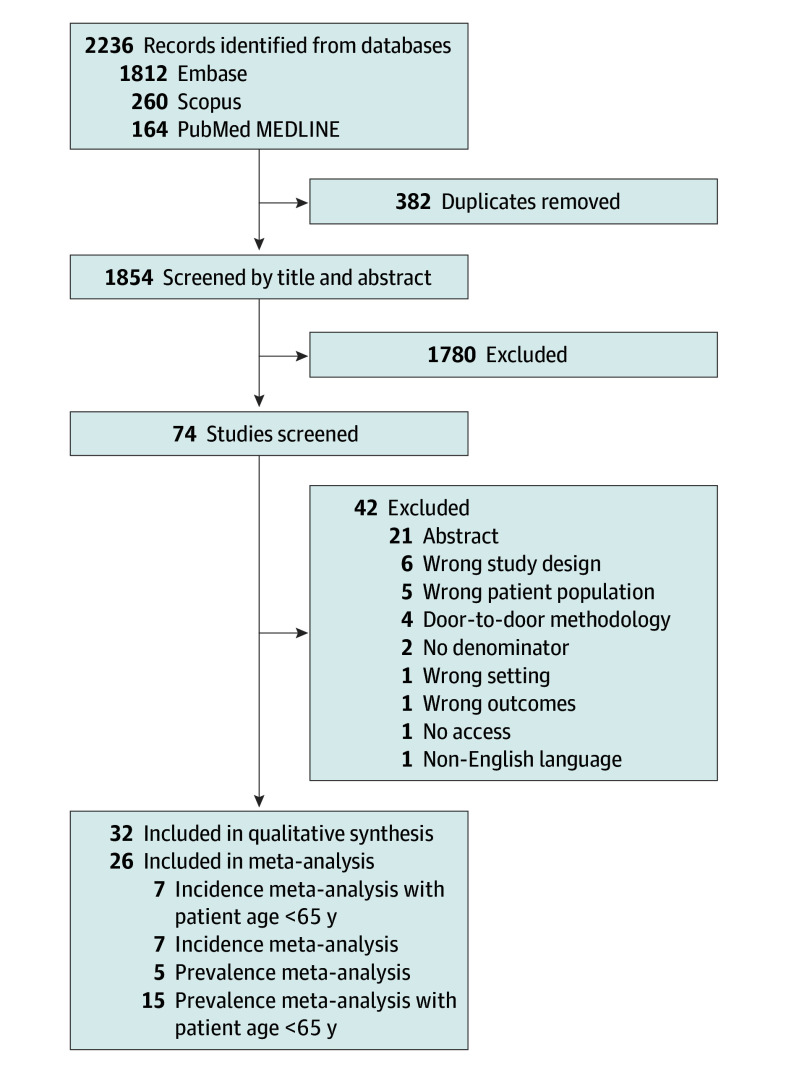
Preferred Reporting Items for Systematic Reviews and Meta-Analyses Guideline Flow Diagram of the Identification, Screening, Eligibility Assessment, and Inclusion of Studies

The search strategy identified 2236 articles, with no additional records found from other sources. After 382 duplicates were removed, 1854 records were screened based on titles and abstracts. Of these, 74 full-text articles were assessed for eligibility, and 42 were excluded for specific reasons. Ultimately, 32 studies^7,18,19,20,21,22,23,24,25,26,27,28,29,30,31,32,33,34,35,36,37,38,39,40,41,42,43,44,45,46,47,48^ met the inclusion criteria (Figure 1). From them, 26 were included in the meta-analyses.^7,18,19,20,21,22,23,24,25,26,27,28,31,33,34,35,37,39,40,42,43,44,45,46,47,48^

### Characteristics of Included Studies

Among the included studies, 12 were on incidence,^7,19,21,22,23,24,30,31,38,41,43,46^ 15 on prevalence,^25,27,28,29,32,33,34,35,36,37,39,42,45,48^ and 5 on both.^18,20,40,44,47^ Eleven were based on the population across all ages,^7,18,19,20,21,22,23,24,25,26,27^ 18 on people younger than 65 years,^18,26,27,28,31,33,34,35,37,39,40,42,43,44,45,46,47,48^ 4 on people 65 years and older,^29,30,32,36,41^ and 2 reported a specific age range not classifiable.^29,30^ The studies were conducted in diverse geographic regions of Europe,^7,18,19,20,21,22,25,26,27,28,29,31,32,34,35,40,41,44,46,47,48^ the United States,^23,24,30,38^ Japan,^33,36,37,42,43^ Australia,^39^ and New Zealand^45^ with target periods spanning between 1990 and 2024. Twenty-one studies consisted of FTD registries,^7,18,19,20,21,22,23,24,25,26,27,28,30,31,34,35,41,44,45,46,47,48^ while 12 consisted of a combination of different population-based study designs.^29,32,33,36,37,38,39,40,42,43,45^ Two studies were conducted at a nationwide level,^43,45^ while all others were restricted to specific regions within their respective countries.

All incidence and combined incidence-prevalence studies followed a registry-based design. For prevalence, 8 studies were based on registries,^25,26,27,28,34,35,45,48^ while 7 used a 2-phase or multistep survey approach.^29,32,33,36,37,39,42^ Diagnostic criteria were mainly Lund-Manchester, Neary, and Rascovsky, except for 2 studies using *ICD* codes,^41,45^ 1 using the *DSM*,^43^ and 1 that did not specify the diagnostic criteria.^42^ Among the 10 studies targeting the population across all ages (6 for incidence, 4 for prevalence, and 1 both), only 5 presented the census data^7,20,21,22,27^; for the other 5,^18,19,23,25,26^ we extracted the census data belonging to the target study period. Overall, the analyzed population included more than 31 million person-years for incidence data and more than 6 million individuals for prevalence data. Detailed characteristics of the studies are listed in eTable 5 in Supplement 1.

### Risk-of-Bias Assessment

All studies measuring FTD incidence showed low risk of bias (eFigure 1 in Supplement 1). Similar results were found for studies estimating FTD prevalence in the whole population only (eFigure 2 in Supplement 1). For those assessing prevalence in people younger than 65 years, about 13% of the studies presented high risk of bias due to issues in the measurement of the condition.^33,37^ Twenty percent of the studies had an uncertain risk of bias.^28,34,42^ For FTD prevalence in individuals 65 years and older, approximately 60% of the studies had a high risk of bias.^29,32,36^ Most of these studies had high attrition rates, issues with the sampling process (participant recruitment), and small sample sizes. The remaining 2 studies had low^27^ and uncertain^34^ risk of bias, respectively.

### Incidence and Prevalence Estimates of FTD and Subtypes

**Figure 2.**
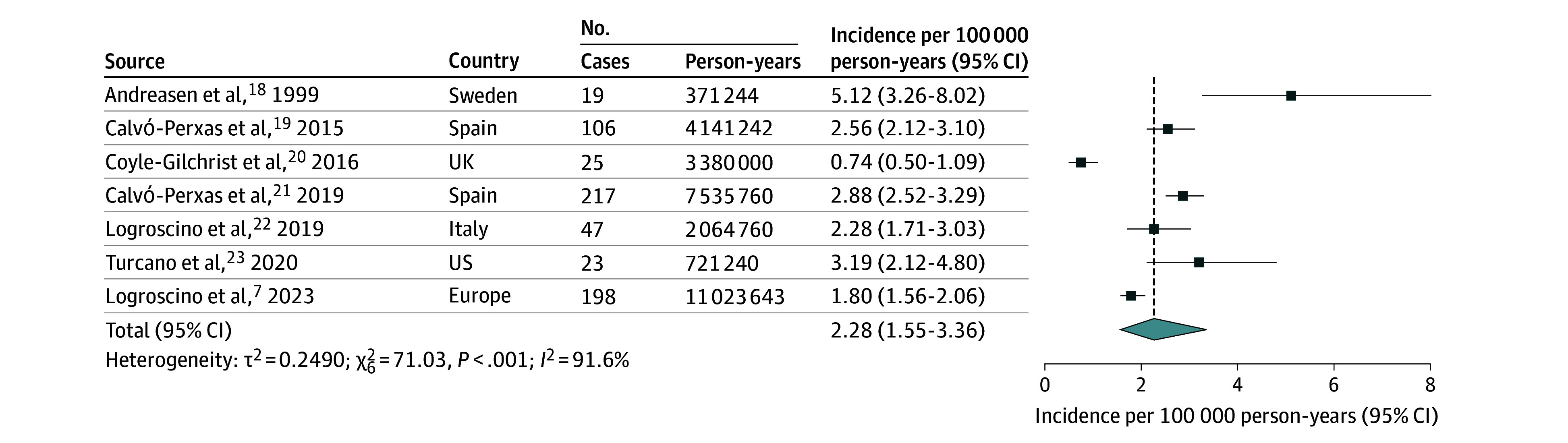
Forest Plot of Pooled Incidence Rate of Frontotemporal Dementia Rate was synthesized across population-based studies using a random-effects model.

**Figure 3.**
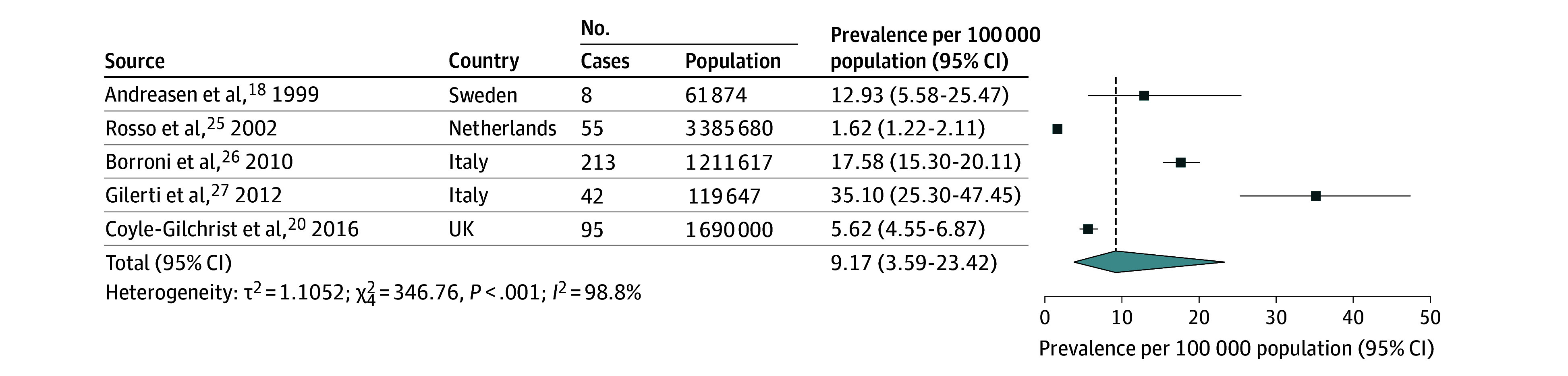
Forest Plot of Pooled Prevalence Rate of Frontotemporal Dementia Rate was synthesized across population-based studies using a random-effects model.

**Figure 4.**
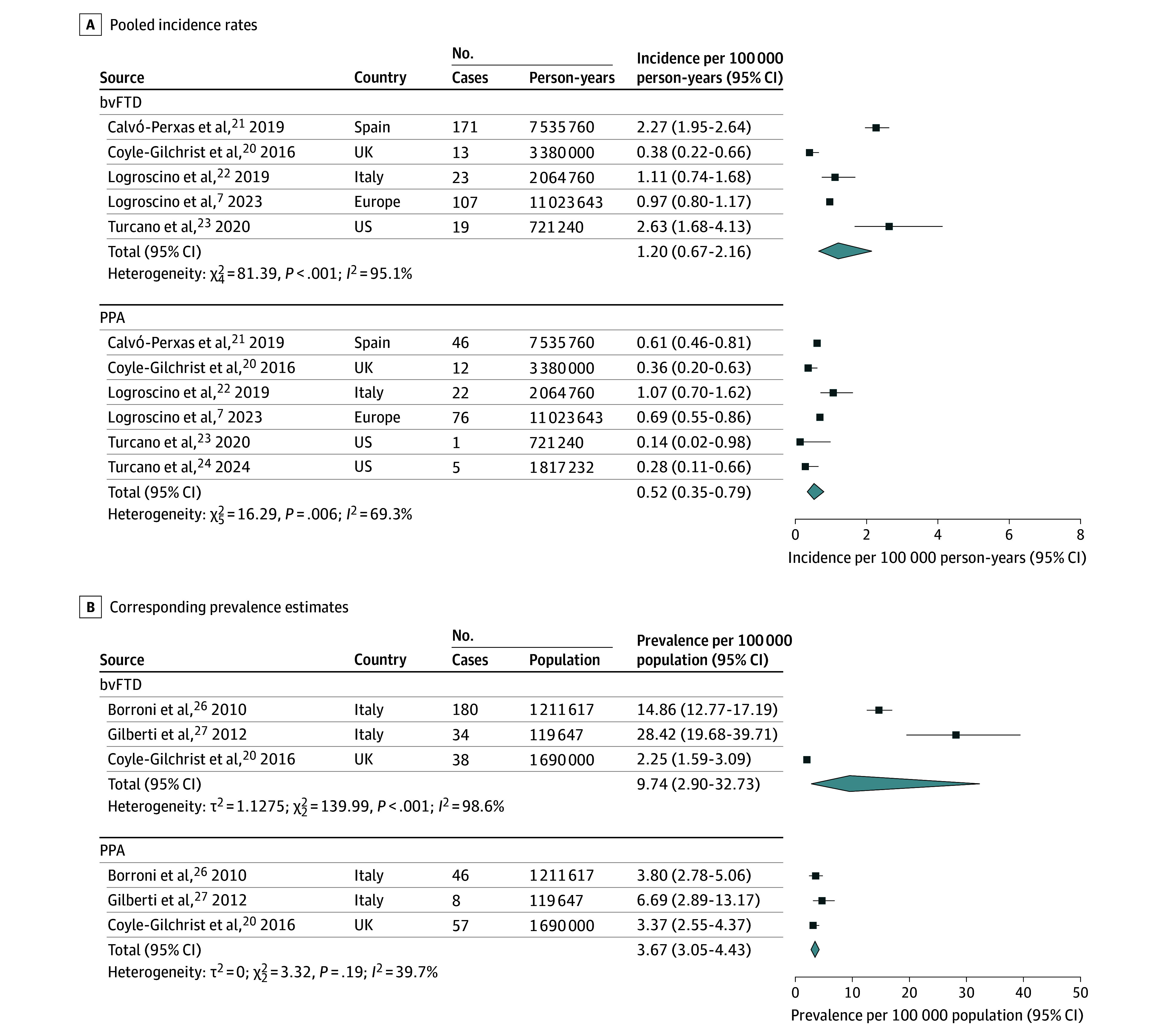
Forest Plot of Incidence and Prevalence for the Behavioral Variant of Frontotemporal Dementia (bvFTD) and Primary Progressive Aphasia (PPA) Between-group heterogeneity was assessed with subgroup analysis.

The pooled incidence rate of FTD was estimated at 2.28 cases per 100 000 person-years (95% CI, 1.55-3.36) (Figure 2). The pooled prevalence of FTD was estimated at 9.17 cases per 100 000 population (95% CI, 3.59-23.42) (Figure 3). Subgroup analyses were performed by FTD subtypes. The pooled incidence rate of bvFTD was 1.20 cases per 100 000 person-years (95% CI, 0.67-2.16) (Figure 4). The pooled incidence rate of PPA was 0.52 cases per 100 000 person-years (95% CI, 0.35-0.79). Among PPA subtypes, nfPPA showed a pooled incidence rate of 0.31 cases per 100 000 person-years (95% CI, 0.15-0.64); svPPA had 0.11 cases per 100 000 person-years (95% CI, 0.06-0.22) (eFigure 3 in Supplement 1). FTD-ALS showed 0.14 cases per 100 000 person-years (95% CI, 0.09-0.22). The pooled prevalence rate of bvFTD was 9.74 cases per 100 000 population (95% CI, 2.90-32.73) (Figure 4). The pooled prevalence of PPA was 3.67 cases per 100 000 population (95% CI, 3.05-4.43). Among PPA subtypes, nfPPA had a prevalence of 2.52 cases per 100 000 population (95% CI, 1.41-4.51), and svPPA had 1.16 cases per 100 000 population (95% CI, 0.83-1.61) (eFigure 4 in Supplement 1).

Heterogeneity across studies was high for FTD overall, with *I^2^* values of 91.6% for incidence and 98.8% for prevalence. However, subgroup analyses revealed notable differences among subtypes: while heterogeneity remained high for bvFTD (*I^2^* for incidence, 95.1%, and prevalence, 98.6%), it was considerably lower for PPA (*I^2^* for incidence, 69.3%, and prevalence, 39.7%). When compared with other major neurodegenerative disorders (eTables 3 and 4 in Supplement 1), FTD showed lower incidence and prevalence than more common conditions such as AD and PD but similar estimates to DLB and higher rates than PSP, CBS, and ALS.

### Incidence and Prevalence of FTD by Age

In populations younger than 65 years, the pooled incidence rate was 1.84 cases per 100 000 person-years (95% CI, 0.79-4.30) (eFigure 5 in Supplement 1). Prevalence rates in populations younger than 65 years were lower, with a pooled prevalence of 7.47 cases per 100 000 population (95% CI, 4.13-13.49) (eFigure 6 in Supplement 1). By contrast, in populations 65 years and older, we were not able to meta-analyze the data because of specific age groups reported by the authors. Two studies reported incidence rates for populations older than 60 years^41^ and 70 years,^38^ respectively (eFigure 7 in Supplement 1). For prevalence, 2 studies reported FTD prevalence for population older than 70 years^32^ and 75 years,^36^ and 1 study for people aged 85 years^29^ (eFigure 8 in Supplement 1). One study^30^ reported incidence rates based on 4 patients with ages from 49 to 69 years, making it too small to be included.

### Sensitivity Analyses

Sensitivity analyses using author-defined denominators confirmed robust estimates. For incidence, the pooled rate was 3.44 cases per 100 000 person-years (95% CI, 1.76-6.73) (eFigure 9 in Supplement 1). For prevalence, the pooled rate was 13.82 cases per 100 000 population (95% CI, 5.27-36.26) (eFigure 10 in Supplement 1). However, the use of author-defined denominators resulted in higher heterogeneity compared with census population data, increasing *I^2^* for incidence from 91.6% to 97.9% and for prevalence from 98.8% to 99.2%. Cumulative meta-analyses demonstrated stable estimates over time, stabilizing at a cumulative pooled incidence rate of 2.09 cases per 100 000 person-years (eFigure 11 in Supplement 1), while the cumulative pooled prevalence stabilized at 9.17 cases per 100 000 population (95% CI, 3.59-23.42) (eFigure 12 in Supplement 1). Leave-1-out analyses showed that no single study significantly influenced the overall pooled estimates for incidence or prevalence. These analyses provided robust pooled rates with consistent confidence intervals (eFigures 13 and 14 in Supplement 1).

## Discussion

Our systematic review and meta-analysis provide the first comprehensive synthesis of incidence and prevalence estimates for FTD and its subtypes. The pooled incidence rate of FTD was 2.28 per 100 000 person-years, while the prevalence was 9.17 per 100 000 population. Heterogeneity across studies was substantial for FTD overall. Subtype analyses revealed that heterogeneity was markedly lower for PPA compared with bvFTD.

Identifying the incidence and prevalence of FTD remains essential to guide clinical research, health service organization, and specialized care delivery.^7^ Although FTD is a rare disorder, reliable epidemiological estimates are crucial to optimize access to diagnostic expertise, support the development of specialized clinical services, and design targeted interventions. The increasing understanding of FTD’s complex pathophysiology, involving molecular subtypes such as FTLD-tau and FTLD with TDP-43 pathology,^49^ highlights the importance of precise data to support advancements in both pharmacological and nonpharmacological treatments. Given the growing pipeline of disease-modifying therapies targeting tau, TDP-43, and genetic mutations like *C9orf72* and *GRN*, accurate epidemiological data are crucial for the design and evaluation of clinical trials.^50^ Furthermore, the significant health care and socioeconomic burden imposed by FTD^51^ underscores the urgency of coordinated efforts to optimize health and social care systems and support translational research aimed at improving outcomes for patients and families.

The observed heterogeneity in bvFTD incidence and prevalence can, in part, be attributed to its symptomatic overlap with primary psychiatric disorders.^52,53^ Early manifestations of bvFTD often mimic conditions such as major depressive disorder, bipolar disorder, and psychotic disorders, leading to frequent misdiagnoses, particularly in the early stages.^53^ Approximately 50% of patients with bvFTD receive a psychiatric diagnosis before their neurodegenerative condition is accurately identified, with diagnostic delays averaging 5 to 6 years.^52^ This overlap is compounded by the phenomenon of bvFTD phenocopies, patients who exhibit behavioral features of bvFTD but lack neuroimaging or pathological evidence of frontotemporal lobar degeneration and remain clinically stable over time.^54^ Phenocopies add further diagnostic uncertainty, blurring the distinction between bvFTD and psychiatric disorders and contributing to the observed heterogeneity in epidemiological estimates. In addition to diagnostic challenges, geographic and genetic factors likely contribute to the heterogeneity in bvFTD incidence and prevalence.^55^ bvFTD is frequently associated with pathogenic variants in genes such as *C9orf72*, *GRN*, and *MAPT*, which show varying distributions across different populations.^56^ These genetic differences may partly explain the variability in bvFTD estimates observed between regions. In contrast, pathogenic variants within the most common dementia-associated genes are rarely implicated in PPA,^57^ resulting in more uniform epidemiological estimates for this subtype. Similarly, although only a limited number of studies have reported population-based estimates for FTD-ALS, available incidence data appear relatively consistent across cohorts.^7,22,23^ While substantially lower than those observed for bvFTD, these estimates are comparable with those reported for individual PPA variants. FTD-ALS represents a clinically and genetically distinct phenotype, most commonly associated with *C9orf72* expansions.^58^ Given its poorer prognosis and shorter survival relative to other FTD subtypes,^59^ further epidemiological research is warranted to better define its frequency and distribution within the population.

Although FTD is a leading cause of early-onset dementia, our findings demonstrate that incidence rates increase with age, peaking in the seventh decade of life. This observation aligns with other neurodegenerative diseases,^60^ reinforcing the need to consider FTD in older adults and not exclusively in younger populations. A multinational European study corroborated this trend, showing a progressive increase in FTD incidence with age.^7^ These findings are crucial for raising awareness among clinicians and informing public health policies that address FTD across the adult lifespan.

To further contextualize our findings, we compared the incidence and prevalence of FTD and its clinical subtypes with estimates available for other major neurodegenerative diseases. While FTD remains less common than AD and PD, its frequency is comparable with DLB and notably higher than that of PSP, CBS, and ALS. These comparisons underscore the burden of FTD within the broader spectrum of neurodegenerative conditions; however, direct comparisons should be interpreted with caution, as age- and sex-standardized estimates were not available across all conditions.

We excluded studies using door-to-door methodologies because of their inherent limitations in capturing sufficient cases of rare neurodegenerative diseases and their susceptibility to underestimating prevalence and incidence.^10,15^ Of note, some of these studies have been conducted in regions with known genetic clusters of FTD,^61^ potentially inflating estimates in localized areas. To ensure consistent and comparable estimates, we also applied census-based population denominators, which reduced heterogeneity and provided more robust estimates within age groups. This methodological improvement addresses prior concerns regarding the arbitrary definition of populations at risk and ensures that estimates are more generalizable across regions and populations.

### Limitations

This meta-analysis has inherent limitations. High heterogeneity reflects the complexity of pooling epidemiological data for a rare and clinically diverse disorder.^55^ Regional genetic variability^56,62^ and potential differences in environmental or modifiable risk factors,^63^ which remain less understood for FTD compared with AD, likely contribute to variability. An additional limitation concerns the classification of PPA variants. While we excluded the logopenic variant because of its strong association with AD pathology, distinguishing PPA subtypes remains difficult in population-based studies given clinical and pathological overlap.^64^ Moreover, primary progressive apraxia of speech (PPAOS), a syndrome not formally classified as PPA but often progressing to nonfluent PPA with CBS or PSP features,^65^ remains largely understudied, with only 1 study to date reporting incidence data.^24^ Future research should aim to report all PPA phenotypes separately and consider including PPAOS cases where appropriate. Furthermore, our analysis was restricted to studies conducted in Europe, the United States, and Japan, limiting the generalizability to other regions and ethnic groups. Efforts to expand data collection to underrepresented populations are critical to addressing this gap.^66^ Additionally, we were unable to summarize estimates stratified by age or sex, as not all studies provided such data, preventing standardized calculations for the European, US, or global populations. To improve future comparability and granularity, population-based studies should report both numerators and denominators stratified by age, sex, and other relevant variables. Nevertheless, we believe that our primary estimates, based on population denominators encompassing all age groups, can serve as a valuable resource for health care planning and policy development.

## Conclusions

Our findings provide robust pooled estimates of the incidence and prevalence of FTD and its subtypes, offering a foundation for future research and public health planning. These data underscore the importance of considering FTD in older adults, enhancing diagnostic approaches, and tailoring health care services to meet the unique needs of patients with this condition. Although FTD is considered a rare disorder, current incidence and prevalence estimate exceeds that of other well-recognized conditions such as PSP, CBS, and ALS. Expanding research efforts to include underrepresented populations will be crucial to better understanding the global burden of FTD and developing effective prevention and treatment strategies tailored to the diverse needs of affected individuals.

## Notes

Supplement 1. **eTable 1.** Search strategy **eTable 2.** List of articles excluded in full-text screening and reasons **eTable 3**. All-age incidence estimates for rare and common neurodegenerative diseases **eTable 4.** All-age prevalence estimates for rare and common neurodegenerative diseases **eTable 5. **Characteristics of Included Studies Reporting Incidence and Prevalence of Frontotemporal Dementia **eFigure 1. **Risk of bias assessment for studies reporting incidence of FTD **eFigure 2. **Risk of bias assessment for studies reporting prevalence of FTD **eFigure 3. **Forest plot of the subgroup analysis for incidence estimates by FTD subtypes **eFigure 4. **Forest plot of the subgroup analysis for prevalence estimates by FTD subtypes **eFigure 5. **Forest plot of incidence estimates for <65 years **eFigure 6. **Forest plot of prevalence estimates for <65 years **eFigure 7. **Forest plot of studies incidence rates for >65 years **eFigure 8. **Forest plot of studies prevalence rates for >65 years **eFigure 9. **Forest plot of sensitivity analysis: incidence estimates using population at risk defined by authors **eFigure 10.** Forest plot of sensitivity analysis: prevalence estimates using population at risk defined by authors **eFigure 11. **Forest plot of cumulative incidence analysis **eFigure 12. **Forest plot of cumulative prevalence analysis **eFigure 13. **Forest plot of leave-one-out analysis for incidence **eFigure 14. **Forest plot of leave-one-out analysis for prevalence

Supplement 2. Data sharing statement
