# Supplementary material for: Incidence and Prevalence of Frontotemporal Dementia: A Systematic Review and Meta-Analysis
Source: JAMA Neurol. 2025 Sep 8;82(11):1144–52. doi: 10.1001/jamaneurol.2025.3307 (PMC12418226; doi:10.1001/jamaneurol.2025.3307)
Supplement: Supplement 1. — eTable 1. Search strategy eTable 2. List of articles excluded in full-text screening and reasons eTable 3. All-age incidence estimates for rare and common neurodegenerative diseases eTable 4. All-age prevalence estimates for rare and common neurodegenerative diseases eTable 5. Characteristics of Included Studies Reporting Incidence and Prevalence of Frontotemporal Dementia eFigure 1. Risk of bias assessment for studies reporting incidence of FTD eFigure 2. Risk of bias assessment for studies reporting prevalence of FTD eFigure 3. Forest plot of the subgroup analysis for incidence estimates by FTD subtypes eFigure 4. Forest plot of the subgroup analysis for prevalence estimates by FTD subtypes eFigure 5. Forest plot of incidence estimates for <65 years eFigure 6. Forest plot of prevalence estimates for <65 years eFigure 7. Forest plot of studies incidence rates for >65 years eFigure 8. Forest plot of studies prevalence rates for >65 years eFigure 9. Forest plot of sensitivity analysis: incidence estimates using population at risk defined by authors eFigure 10. Forest plot of sensitivity analysis: prevalence estimates using population at risk defined by authors eFigure 11. Forest plot of cumulative incidence analysis eFigure 12. Forest plot of cumulative prevalence analysis eFigure 13. Forest plot of leave-one-out analysis for incidence eFigure 14. Forest plot of leave-one-out analysis for prevalence [file jamaneurol-e253307-s001.pdf]

Supplemental Online Content

Urso D, Giannoni-Luza S, Brayne C, Ray N, Logroscino G, et al. Incidence and prevalence of frontotemporal dementia: a systematic review and meta-analysis. *JAMA Neuro*. Published online September 8, 2025. doi:10.1001/jamaneurol.2025.3307

eTable 1. Search strategy
eTable 2. List of articles excluded in full-text screening and reasons
eTable 3. All-age incidence estimates for rare and common neurodegenerative diseases
eTable 4. All-age prevalence estimates for rare and common neurodegenerative diseases
eTable 5. Characteristics of Included Studies Reporting Incidence and Prevalence of Frontotemporal Dementia
eFigure 1. Risk of bias assessment for studies reporting incidence of FTD
eFigure 2. Risk of bias assessment for studies reporting prevalence of FTD
eFigure 3. Forest plot of the subgroup analysis for incidence estimates by FTD subtypes
eFigure 4. Forest plot of the subgroup analysis for prevalence estimates by FTD subtypes
eFigure 5. Forest plot of incidence estimates for <65 years
eFigure 6. Forest plot of prevalence estimates for <65 years
eFigure 7. Forest plot of studies incidence rates for >65 years
eFigure 8. Forest plot of studies prevalence rates for >65 years
eFigure 9. Forest plot of sensitivity analysis: incidence estimates using population at risk defined by authors
eFigure 10. Forest plot of sensitivity analysis: prevalence estimates using population at risk defined by authors
eFigure 11. Forest plot of cumulative incidence analysis
eFigure 12. Forest plot of cumulative prevalence analysis
eFigure 13. Forest plot of leave-one-out analysis for incidence
eFigure 14. Forest plot of leave-one-out analysis for prevalence

This supplemental material has been provided by the authors to give readers additional information about their work.

© 2025 Urso D et al. *JAMA Neurology*.

eTable 1. Search strategy.

Database	Search Terms	Results
**MEDLINE**<br><br>**Pubmed**<br><br>**22.10.24**	("frontotemporal lobar degeneration"[MeSH Terms] OR "frontotemporal dementia behavioral variant"[Title/Abstract] OR "Behavioral variant frontotemporal dementia"[Title/Abstract] OR "aphasia, primary progressive"[MeSH Terms] OR "Primary progressive aphasia"[Title/Abstract] OR "Primary Progressive Nonfluent Aphasia"[Title/Abstract] OR "semantic aphasia"[Title/Abstract] OR "logopenic aphasia"[Title/Abstract]) AND ("prevalence"[MeSH Terms] OR "incidence"[MeSH Terms] OR "incidence"[Title/Abstract] OR "incidence"[Title/Abstract] OR "population based study"[Title/Abstract])	164
**EMBASE**<br><br>**22.10.24**	('frontotemporal dementia'/exp OR 'frontotemporal dementia' OR 'frontotemporal lobe degeneration' OR 'frontotemporal dementia behavioral variant' OR 'frontal variant frontotemporal dementia' OR 'primary progressive aphasia' OR 'progressive nonfluent aphasia' OR 'logopenic progressive aphasia' OR 'semantic aphasia') AND ('incidence' OR 'prevalence' OR 'population based study')	1812
**SCOPUS**<br><br>**22.10.24**	(TITLE-ABS ("frontotemporal lobar degeneration") OR TITLE-ABS ("frontotemporal dementia behavioral variant") OR TITLE-ABS ("behavioral variant frontotemporal dementia") OR TITLE-ABS ("primary progressive aphasia") OR TITLE-ABS ("primary progressive nonfluent aphasia") OR TITLE-ABS ("semantic aphasia") OR TITLE-ABS ("logopenic aphasia")) AND (TITLE-ABS ("prevalence") OR TITLE-ABS ("incidence") OR TITLE-ABS ("population-based study"))	260

© 2025 Urso D et al. *JAMA Neurology*.

eTable 2. List of articles excluded in full-text screening and reasons.

Order	Title	Author and year	Reason of exclusion
1	Is Frontotemporal Lobar Degeneration a rare disorder? evidence from a population-based study in Brescia county, Italy	Alberici 2009	Abstract
2	Epidemiology and genetics of frontotemporal dementia: A door-to-door survey in Southern Italy	Bernardi 2012	Wrong patient population
3	Epidemiology of frontotemporal dementia in southern Italy	Bruni 2009	Abstract
4	The population based prevalence and phenotype of 9p21 hexanucleotide repeats in ALS/FTD	Byrne 2012	Abstract
5	The incidence of FTLD in salento: Preliminary results from Puglia FTD registry	Capozzo 2018	Abstract
6	The prevalence of frontotemporal dementia and progressive supranuclear palsy the UK: Preliminary data from the PiPPIN Study	Coyle-Gilchrist 2014	Abstract
7	The genetic and epidemiology of frontotemporal dementia in Sardinia	Di Stefano 2016	Abstract
8	Parkinsonism in frontotemporal dementia: Data from a Sardinian cohort	Di Stefano 2016	Abstract
9	Epidemiology of early onset dementia in Northern Italy	Fiondella 2020	Abstract
10	Frontotemporal lobar degeneration (FTLD) in an isolated population	Gilberti 2010	Abstract
11	Estimates of frontotemporal dementia by geographic regions	Gillis 2021	Abstract
12	Frontotemporal dementia in the Marche region: An attempt to estimate incidence and prevalence	Girelli 2016	Abstract
13	Effect of diagnostic criteria on prevalence of frontotemporal dementia in the elderly	Gislason 2015	Wrong study design
14	Prevalence and Determinants of Diagnosed Dementia: A Registry Linkage Study Linking Diagnosis of Dementia in the Population-Based HUNT Study to Registry Diagnosis of Dementia in Primary Care and Hospitals in Norway	Gjøra 2024	Wrong outcomes
15	Prevalence of dementia subtypes in United States Medicare fee-for-service beneficiaries, 2011–2013	Goodman 2017	Wrong patient population
16	Global incidence of young-onset dementia: A systematic review and meta-analysis	Hendriks 2023	Wrong study design
17	Trends in the prevalence of dementia in Japan: A preliminary report	Ikejima 2011	Abstract
18	Prevalence and illnesses causing early-onset dementia in Japan: A multi-center study	Ikejima 2009	Abstract
19	Estimating the number of persons with frontotemporal lobar degeneration in the US population	Knopman 2011	Wrong study design
20	Frequency and clinical characteristics of the individuals with presenile dementia in Aichi prefecture	Konagaya 2009	Language
21	Epidemiology of frontotemporal degeneration in a large de-identified database Alan	Lerner 2012	Abstract

© 2025 Urso D et al. *JAMA Neurology*.

22	Incidence of syndromes associated with Frontotemporal Lobar Degeneration	Logroscino 2023	Abstract
23	The clinical incidence of frontal dementia	López-Pousa 2002	Wrong setting
24	Ten-Year Prevalence of Cognitive Impairment Diagnoses and Associated Medical and Psychiatric Conditions in a National Cohort of Older Female Veterans	Lwi 2019	Wrong patient population
25	PREVALENCE OF DEMENTIA AND ASSOCIATED CONDITIONS IN A NATIONAL COHORT OF OLDER FEMALE VETERANS	Lwi 2018	Abstract
26	Prevalence and Ascertainment of Dementia Cases in the Malmö Diet and Cancer Study	Nägga 2022	Wrong study design
27	Age-related incidence and family history in Frontotemporal dementia: Data from the Swedish Dementia Registry	Nilson 2014	No denominator
28	A unique common ancestor introduced P301L mutation in MAPT gene in frontotemporal dementia patients from Barcelona (Baix Llobregat, Spain)	Palencia-Madrid 2019	Wrong study design
29	Identification of the causative gene of FTLD in a southern Italian isolated population	Puca 2012	Abstract
30	Prevalence of dementia in urban and regional Aboriginal Australians	Radford 2015	Wrong patient population
31	HELIAD study: Dementia prevalence in Greece	*Sakka 2017*	Abstract
32	Prevalence rates of early onset Alzheimer's disease and fronto-temporal dementia clinical phenotypes among age groups in the Province of Modena, Italy	Salemme 2021	Abstract
33	C9orf72-associated frontotemporal dementia in the Russian population	Shpilyujova 2020	Wrong study design
34	New cases of dementia are rising in elderly populations in Wales, UK	Stevenson-Hoare 2023	No denominator
35	Prevalence of Dementia and Subtypes in Valladolid, Northwestern Spain: The DEMINVALL Study	Tola-Arribas 2013	Door-to-door
36	Prevalence of different types of dementia in a population based study in Southern Italy: The GreatAge study	Tortelli 2014	Abstract
37	Incidence and trends of frontotemporal disorders in olmsted county: A population based study (1995-2010)	Turcano 2019	Abstract
38	Differences among dementias according to onset age: Study based on dementia registry data	Vilalta-Franch 2008	No access
39	Prevalence of dementia in the rural island town of Ama-cho, Japan	Wada-Isoe 2009	Door-to-door
40	Prevalence of alzheimer's disease, vascular dementia and dementia with Lewy bodies in a Japanese population	Yamada 2001	Door-to-door
41	Prevalence of dementia in the older Japanese-Brazilian population	Yamada 2002	Wrong patient population
42	Prevalence of dementia and dementia subtypes among community-dwelling elderly people in northern Nigeria	Yusuf 2011	Door-to-door

© 2025 Urso D et al. *JAMA Neurology*.

eTable 3. All-age incidence estimates for rare and common neurodegenerative diseases

Neurodegenerative disorder	Author, Year	Study Type	Estimate	95% CI
**FTD**	-	Meta-analysis	2.28	1.55 – 3.36
bvFTD	-	Meta-analysis	1.20	0.67 – 2.16
PPA	-	Meta-analysis	0.52	0.35 – 0.79
**PSP**	Lyons et al., 2023[1]	Meta-analysis	0.81	0.48 – 1.37
**CBS**	Lyons et al., 2023[1]	Meta-analysis	0.28	0.15 – 0.52
**ALS**	Xu et al., 2020[2]	Meta-analysis	1.59	1.39 – 1.81
**DLB***	Savica et al., 2013[3]	Reconstructed cohort study	3.5	-
**PD†**	Hirsch et al., 2016[4]	Meta-analysis	37.55	-
**AD‡**	Kukull et al., 2002[5]	Prospective cohort study	1426	1220 – 1670

Estimates represent crude all-age incidence rates per 100,000 person-years, derived from meta-analyses of population-based studies. When meta-analyses were not available, the most recent and methodologically robust single studies were included. Abbreviations: FTD, Frontotemporal Dementia; bvFTD, Behavioral Variant Frontotemporal Dementia; PPA, Primary Progressive Aphasia; PSP, Progressive Supranuclear Palsy; CBS, Corticobasal Syndrome; ALS, Amyotrophic Lateral Sclerosis; DLB, Dementia with Lewy Bodies; PD, Parkinson's Disease; AD, Alzheimer's Disease.

*DLB estimate from *Savica et al.,* 2013 is derived from a population-based reconstructed cohort study using the Rochester Epidemiology Project in Olmsted County, Minnesota; no all-age meta-analysis is currently available.

†PD estimate from *Hirsch et al.,* 2016 refers to individuals aged ≥40 years; no all-age meta-analysis available.

‡AD estimate from *Kukull et al*., 2002 refers to individuals aged ≥65 years; no all-age meta-analysis or study available.

1. Lyons, S., Trépel, D., Lynch, T., Walsh, R., & O'Dowd, S. (2023). The prevalence and incidence of progressive supranuclear palsy and corticobasal syndrome: a systematic review and meta-analysis. Journal of neurology, 270(9), 4451–4465. https://doi.org/10.1007/s00415-023-11791-2
2. Xu, L., Liu, T., Liu, L., Yao, X., Chen, L., Fan, D., Zhan, S., & Wang, S. (2020). Global variation in prevalence and incidence of amyotrophic lateral sclerosis: a systematic review and meta-analysis. Journal of neurology, 267(4), 944–953. https://doi.org/10.1007/s00415-019-09652-y
3. Savica R, Grossardt BR, Bower JH, Boeve BF, Ahlskog JE, Rocca WA. Incidence of dementia with Lewy bodies and Parkinson disease dementia. JAMA Neurol. 2013 Nov;70(11):1396-402. doi: 10.1001/jamaneurol.2013.3579. PMID: 24042491; PMCID: PMC4181848.
4. Hirsch, L., Jette, N., Frolkis, A., Steeves, T., & Pringsheim, T. (2016). The Incidence of Parkinson's Disease: A Systematic Review and Meta-Analysis. Neuroepidemiology, 46(4), 292–300. https://doi.org/10.1159/000445751
5. Kukull, W. A., Higdon, R., Bowen, J. D., McCormick, W. C., Teri, L., Schellenberg, G. D., van Belle, G., Jolley, L., & Larson, E. B. (2002). Dementia and Alzheimer disease incidence: a prospective cohort study. Archives of neurology, 59(11), 1737–1746. https://doi.org/10.1001/archneur.59.11.1737

© 2025 Urso D et al. *JAMA Neurology*.

eTable 4. All-age prevalence estimates for rare and common neurodegenerative diseases

Neurodegenerative disorder	Author, Year	Study Type	Estimate	95% CI
**FTD**	-	Meta-analysis	9.17	3.59 – 23.42
**bvFTD**	-	Meta-analysis	9.74	2.90 – 32.73
**PPA**	-	Meta-analysis	3.67	3.05 – 4.43
**PSP**	Lyons et al., 2023[1]	Meta-analysis	6.92	4.33 – 11.06
**CBS**	Lyons et al., 2023[1]	Meta-analysis	3.91	2.03 – 7.51
**ALS**	Xu et al., 2020[2]	Meta-analysis	4.42	3.92 – 4.96
**PD**	Zhu et al., 2024[3]	Meta-analysis	151	119 – 188
**AD***	Leahy et al., 2023[4]	Retrospective observational study	378.39	359.36 – 398.44

Estimates represent crude all-age prevalence rates per 100,000 persons, derived from meta-analyses of population-based studies. When meta-analyses were not available, the most recent and methodologically robust single studies were included. Abbreviations: FTD, Frontotemporal Dementia; bvFTD, Behavioral Variant Frontotemporal Dementia; PPA, Primary Progressive Aphasia; PSP, Progressive Supranuclear Palsy; CBS, Corticobasal Syndrome; ALS, Amyotrophic Lateral Sclerosis; DLB, Dementia with Lewy Bodies; PD, Parkinson’s Disease; AD, Alzheimer’s Disease.
*AD estimate from *Leahy et al*. 2023 is derived from a retrospective observational study based on electronic health records from the Clinical Practice Research Datalink (CPRD) in England; no all-age meta-analysis is currently available.

1. Lyons, S., Trépel, D., Lynch, T., Walsh, R., & O'Dowd, S. (2023). The prevalence and incidence of progressive supranuclear palsy and corticobasal syndrome: a systematic review and meta-analysis. Journal of neurology, 270(9), 4451–4465. https://doi.org/10.1007/s00415-023-11791-2
2. Xu, L., Liu, T., Liu, L., Yao, X., Chen, L., Fan, D., Zhan, S., & Wang, S. (2020). Global variation in prevalence and incidence of amyotrophic lateral sclerosis: a systematic review and meta-analysis. Journal of neurology, 267(4), 944–953. https://doi.org/10.1007/s00415-019-09652-y
3. Zhu, J., Cui, Y., Zhang, J., Yan, R., Su, D., Zhao, D., Wang, A., & Feng, T. (2024). Temporal trends in the prevalence of Parkinson's disease from 1980 to 2023: a systematic review and meta-analysis. The lancet. Healthy longevity, 5(7), e464–e479. https://doi.org/10.1016/S2666-7568(24)00094-1
4. Leahy, T. P., Simpson, A., Sammon, C., Ballard, C., & Gsteiger, S. (2023). Estimating the prevalence of diagnosed Alzheimer disease in England across deprivation groups using electronic health records: a clinical practice research datalink study. BMJ open, 13(10), e075800. https://doi.org/10.1136/bmjopen-2023-075800

eTable 5. Characteristics of Included Studies Reporting Incidence and Prevalence of Frontotemporal Dementia

Study ID, year of publication	Geographically defined area,	Study design	Target Period	Diagnostic Criteria	Age group	Age range	Frequency Measure
Andreasen, 1999[18]	Piteå River Valley, Sweden	Registry	1990-1995	Lund-Manchester	All and early onset	42-92	Incidence and Prevalence
Ratnavalli, 2002[28]	Cambridgeshire Health Authority, UK	Registry	2000	Neary	Early-onset	<65	Prevalence
Gislason, 2003[29]	Gothenburg, Sweden	Two-phases survey	1986-1987	Lund-Manchester	Specific	85	Prevalence
Rosso, 2003[25]	Zuid-Holland, The Netherlands	Registry	1994	Neary, Lund-Manchester	All	30-79	Prevalence
Knopman, 2004[30]	Rochester, MN, USA	Registry	1990-1994	Neary and McKhan	Specific	49-69	Incidence
Mercy, 2008[31]	Cambridgeshire, UK	Registry	2000-2006	Neary	Early-onset	<65	Incidence
Gascón-Bayarri, 2007[32]	El Prat de Llobregat, Catalonia, Spain	Two-phases survey	2001	Neary	Late-onset	>70	Prevalence
Ikejima, 2009[33]	Ibaraki Prefecture, Japan	Two-phases survey	2006	Lund-Manchester	Early-onset	20-64	Prevalence
Borroni, 2010[26]	Brescia province, Italy	Registry	2001	Neary and McKahnn	All, and early-onset	All	Prevalence
Garre-Olmo, 2010[34]	Girona, Catalonia, Spain	Registry	2007-2009	Lund-Manchester, Neary and Messulam.	Early-onset	30-64	Prevalence
Borroni, 2011[35]	Brescia province, Italy	Registry	2009	Neary and McKhann	Early-onset	45-65	Prevalence
Gilberti, 2012[27]	Vallecamonica, Esine Brescia, Italy	Registry	2010	Neary and McKahn	All, and early-onset	All	Prevalence
Meguro, 2012[36]	Kurihara, Japan	Two-phases survey	2010	Lund-Manchester	Late-onset	>75	Prevalence
Ikejima, 2014[37]	Ibaraki, Gunma, Toyama, Ehime, and Kunamoto, Japan	Two-phases survey	2006	Lund-Manchester	Early-onset	18-64	Prevalence
Knopman, 2014[38]	Olmsted County, Minnesota, USA	Registry	2004-2013	Neary	Late-onset	70-89	Incidence

© 2025 Urso D et al. *JAMA Neurology*.

Withhall, 2014[39]	Eastern Sydney, Australia	Three-phases survey	2007-2008	Lund-Manchester	Early-onset	30-64	Prevalence
Calvó-Perxas, 2015[19]	Girona, Catalunia, Spain	Registry	2007-2012	Neary, Lund-Manchester	All	>55	Incidence
Luukkainen, 2015[40]	Northern Ostrobothnia, Finland	Registry	2006-2010	Neary, Rascovsky and Gorno Tempini	Early-onset	45-64	Incidence, and prevalence
Coyle-Gilchrist, 2016[20]	Cambridgeshire and Norfolk, UK	Registry	2013-2014	Rascovsky, Gorno-tempini	All	All	Incidence, and prevalence
Calvó-Perxas, 2019[21]	Girona, Catalunia, Spain	Registry	2007-2016	Neary, Lund-Manchester	All	30-101	Incidence
Logroscino, 2019[22]	Salento and Brescia, Italy	Registry	2017	Rascovsky, Gorno-tempini	All	All	Incidence
Petersen, 2019[41]	Faroe Islands, Denmark	Registry	2010-2017	ICD	Late-onset	>60	Incidence
Awata, 2020[42]	12 of Japan's 47 prefectures	Two-phases survey	2018	Not defined	Early-onset	18-64	Prevalence
Edahiro, 2020[43]	Japan	Registry	2018-2019	DSM-V	Early-onset	18-64	Incidence
Turcano, 2020[23]	Olmsted County, Minnsesota, USA	Registry	1995-2010	Rascovsky, Gorno-tempini	All	40-100	Incidence
Chiari, 2021[44]	Modena, Italy	Registry	2006-2019	Rascovsky, Gorno-tempini	Early-onset	30-64	Incidence, and prevalence
Ryan, 2022[45]	New Zealand	Registry	2016-2020	ICD	Early-onset	0-64	Prevalence
Logroscino, 2023[7]	Different areas in Europe†	Registry	2018-2019	Rascovsky, Gorno-tempini	All	0-120	Incidence
Borroni, 2024[46]	Brescia province, Italy	Registry	2019	Rascovsky and Gorno-Tempini	Early-onset	30-65	Incidence
Krüger, 2024[47]	Northern Ostrobothnia and Northern Savonia Provinces, Finland	Registry	2010-2021	Rascovsky and Gorno-Tempini	Early-onset	30-64	Incidence, and prevalence
Turcano 2024†[24]	Olmsted County, Minnsesota, USA	Registry	2011-2022	Gorno-Tempini	All	>18	Incidence
Zamboni, 2024[48]	Modena, Italy	Registry	2019	Rascovsky and Gorno-Tempini	Early-onset	40-65	Prevalence

† Bulgaria, Finland, Germany, Italy, Serbia, Spain, Sweden, The Netherlands, and UK.

© 2025 Urso D et al. *JAMA Neurology*.

eFigure 1. Risk of bias assessment for studies reporting incidence of FTD.

	Representativeness of the exposed cohort	Selection of the non-exposed cohort	Ascertainment of exposure	Outcome of interest not present at baseline	Comparability	Outcome Assessment	Adequacy of follow-up duration	Adequacy of follow up of cohorts	Total score	Quality
**ALL**										
Andreasen 1999 [15]	★	★	★	★	Not Applied	★	★	★	7	Good
Calvó-Perxas 2015 [16]	★	★	★	★		★	★	★	7	Good
Coyle-Gilchrist 2016 [17]	★	★	★	★		★	★	★	7	Good
Calvó-Perxas 2019 [18]	★	★	★	★		★	★	★	7	Good
Logroscino 2019 [19]	★	★	★	★		★	★	★	7	Good
Turcano 2020 [20]	★	★	★	★		★	★	★	7	Good
Logroscino 2023 [7]	★	★	★	★		★	★	★	7	Good
**<65**										
Andreasen 1999 [15]	★	★	★	★		★	★	★	7	Good
Mercy 2008 [28]	★	★	★	★	Not Applied	★	★	★	7	Good
Luukkainen 2015 [37]	★	★	★	★		★	★	★	7	Good
Edahiro 2020 [40]	★	★	★	★		★	★	★	7	Good
Chiari 2021 [41]	★	★	★	★		★	★	★	7	Good
Borroni 2024 [23]	★	★	★	★		★	★	★	7	Good
Krüger 2024 [44]	★	★	★	★		★	★	★	7	Good
**≥65**										
Knopman 2014 [35]	★	★	★	★	Not Applied	★	★	★	7	Good
Petersen 2019 [38]	★	★	★	★		★	★	★	7	Good
**Specific**										
Knopman 2004 [27]	★	★	★	★	NA	★	★	★	7	Good

© 2025 Urso D et al. *JAMA Neurology*.

eFigure 2. Risk of bias assessment for studies reporting prevalence of FTD.

Prevalence: Joanna Briggs Institute Checklist

	Target population	Sampling process	Sample Size	Subjects and Setting Description	Data coverage data anaylsis	Ascertainment of Condiiton	Measurement of Condition	Appropriate Analysis	Response rate	Overall
**ALL**										
Andreasen 1999 [15]	+	+	+	+	+	+	+	+	+	+
Rosso 2003 [22]	+	+	+	+	+	+	+	+	+	+
Borroni 2010 [23]	+	+	+	+	+	+	+	+	+	+
Coyle-Gilchrist 2016 [17]	+	+	+	+	+	+	+	+	+	+
Gilberti 2012 [24]	+	+	+	+	+	+	+	+	+	+
**< 65**										
Andreasen 1999 [15]	+	+	+	+	+	+	+	+	+	+
Ratnavali 2002 [25]	+	+	+	+	+	+	+	?	+	?
Ikejima 2009 [30]	+	+	+	+	+	+	-	+	+	-
Borroni 2010 [23]	+	+	+	+	+	+	+	+	+	+
Garre-Olmo 2010 [31]	+	+	+	+	+	+	?	+	+	?
Borroni 2011 [32]	+	+	+	+	+	+	+	+	+	+
Gilberti 2012 [24]	+	+	+	+	+	+	+	+	+	+
Ikejima 2014 [34]	+	+	+	+	+	+	-	+	+	-
Withall 2014 [36]	+	+	+	+	+	+	+	+	+	+
Luukkainen 2015 [37]	+	+	+	+	+	+	+	+	+	+
Awata 2020 [39]	+	+	+	+	?	?	?	+	?	?
Chiari 2021 [41]	+	+	+	+	+	+	+	+	+	+
Ryan 2022 [42]	+	+	+	+	+	+	+	+	+	+
Krüger 2024 [44]	+	+	+	+	+	+	+	+	+	+
Zamboni 2024 [45]	+	+	+	+	+	+	+	+	+	+
**≥ 65**										
Gislason 2003 [26]	+	+	-	+	?	+	+	+	-	-
Gascón-Bayarri 2007 [29]	+	-	-	+	+	+	-	+	-	-
Garre-Olmo 2010 [31]	+	+	+	+	+	+	?	+	+	?
Gilberti 2012 [24]	+	+	+	+	+	+	+	+	+	+
Meguro 2012 [33]	+	-	?	+	-	+	+	+	-	-

© 2025 Urso D et al. *JAMA Neurology*.

eFigure 3. Forest plot of the subgroup analysis for incidence estimates by FTD subtypes.

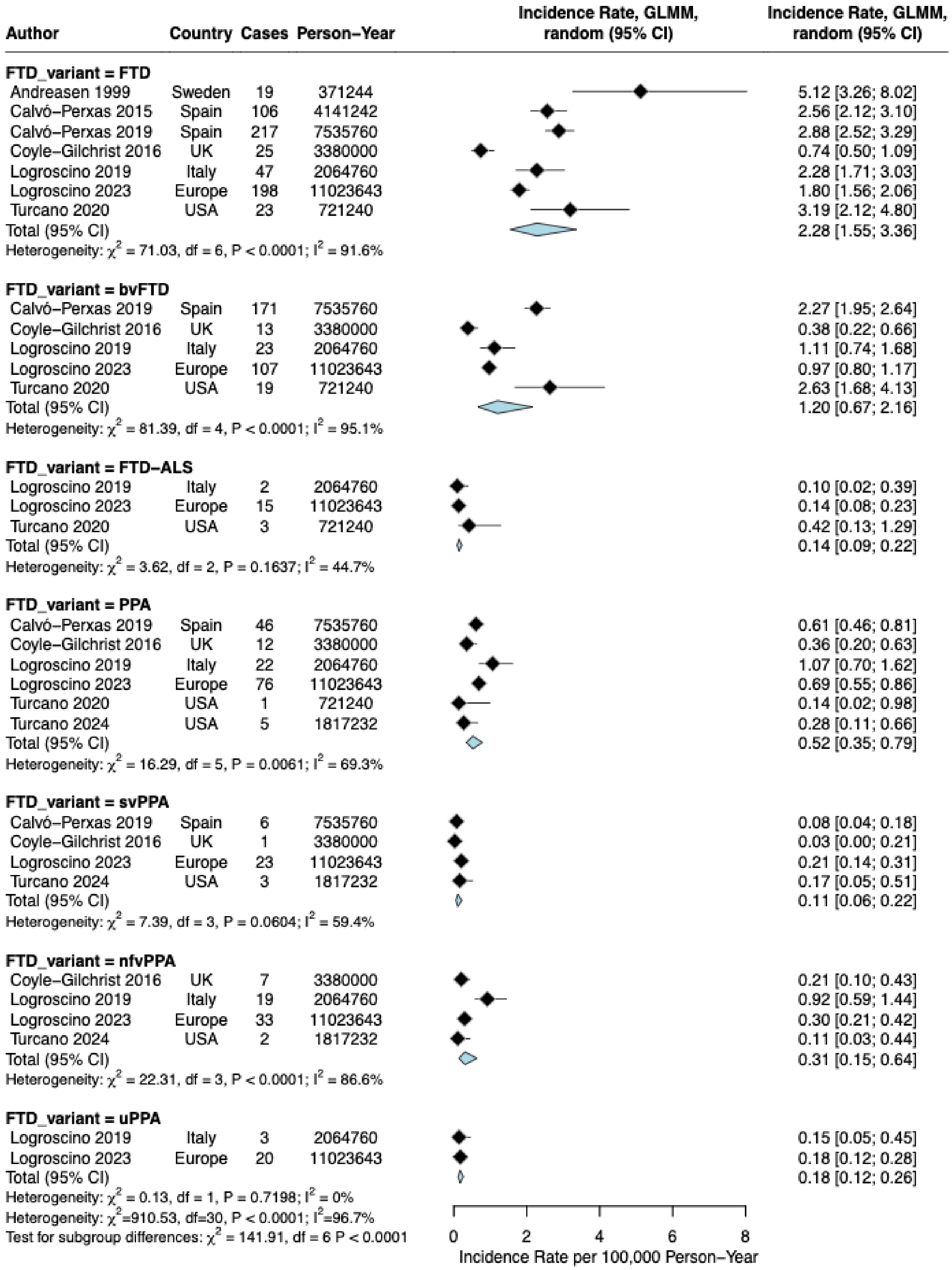

© 2025 Urso D et al. *JAMA Neurology*.

eFigure 4. Forest plot of the subgroup analysis for prevalence estimates by FTD subtypes

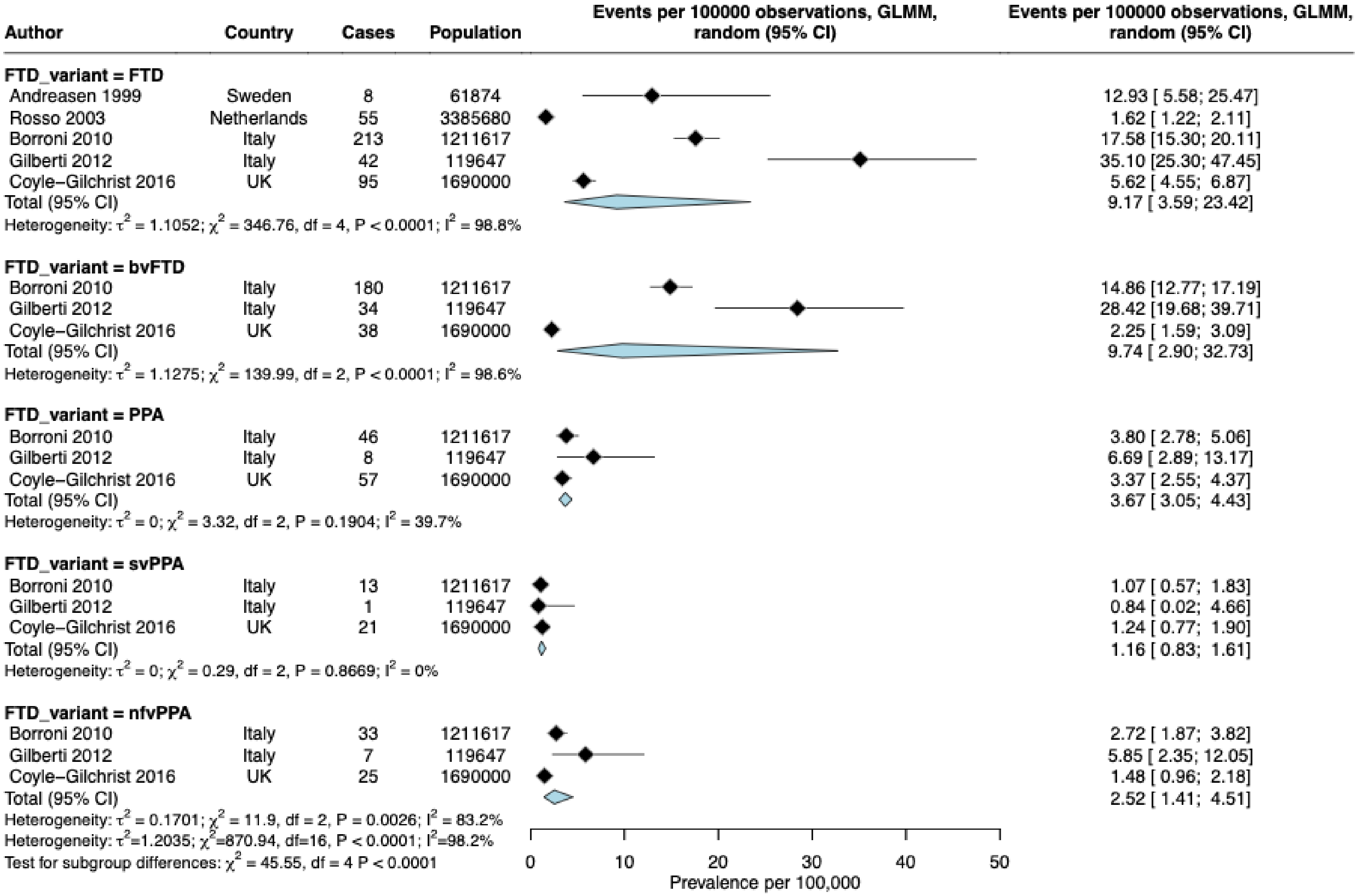

© 2025 Urso D et al. *JAMA Neurology*.

eFigure 5. Forest plot of incidence estimates for <65 years.

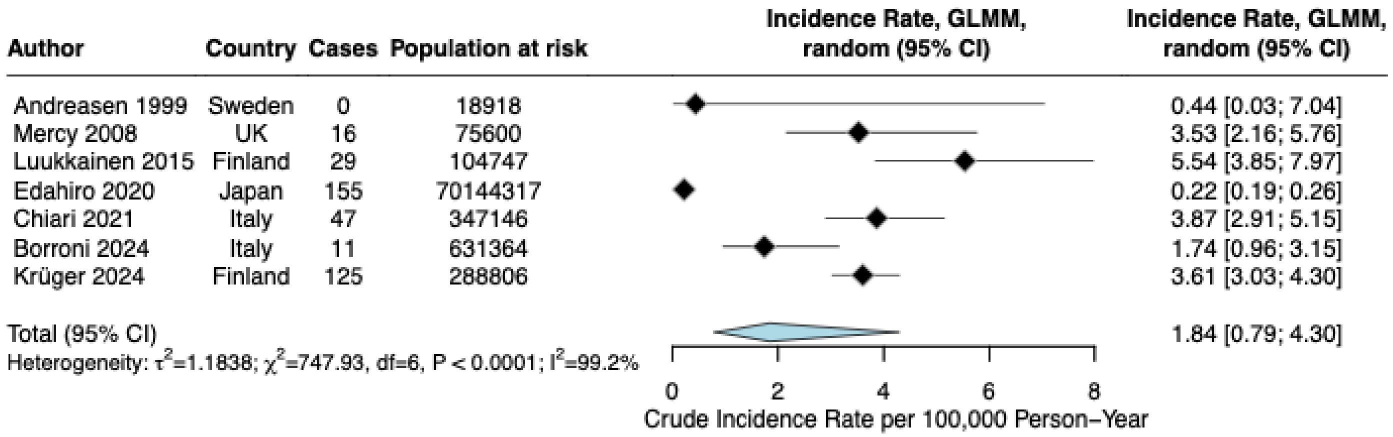

© 2025 Urso D et al. *JAMA Neurology*.

eFigure 6. Forest plot of prevalence estimates for <65 years.

Author	Country	Cases	Population	Design	Events per 100000 observations, GLMM, random (95% CI)
Andreasen 1999	Sweden	0	18918	Registry	0.00 [0.00; 19.50]
Ratnavalli 2002	UK	11	72815	Registry	15.11 [7.54; 27.03]
Ikejima 2009	Japan	18	1799340	Two−step	1.00 [0.59; 1.58]
Borroni 2010	Italy	72	322334	Registry	22.34 [17.48; 28.13]
Garre−Olmo 2010	Spain	70	690207	Registry	10.14 [7.91; 12.81]
Borroni 2011	Italy	94	317107	Registry	29.64 [23.96; 36.27]
Gilberti 2012	Italy	10	31703	Registry	31.54 [15.13; 58.00]
Ikejima 2014	Japan	74	5664741	Two−step	1.31 [1.03; 1.64]
Withall 2014	Australia	7	129070	Three−steps	5.42 [2.18; 11.17]
Luukkainen 2015	Finland	22	341164	Registry	6.45 [4.04; 9.76]
Awata 2020	Japan	383	11630322	Two−step	3.29 [2.97; 3.64]
Chiari 2021	Italy	68	347146	Registry	19.59 [15.21; 24.83]
Ryan 2022	Italy	78	4169754	Nationwide	1.87 [1.48; 2.33]
Krüger 2024	Finland	39	280828	Registry	13.89 [9.88; 18.98]
Zamboni 2024	Italy	66	265281	Registry	24.88 [19.24; 31.65]
Total (95% CI)					7.47 [4.13; 13.49]

Heterogeneity: $\tau^2=1.3004$; $\chi^2=1147.93$, df=14, $P < 0.0001$; $I^2=98.8\%$

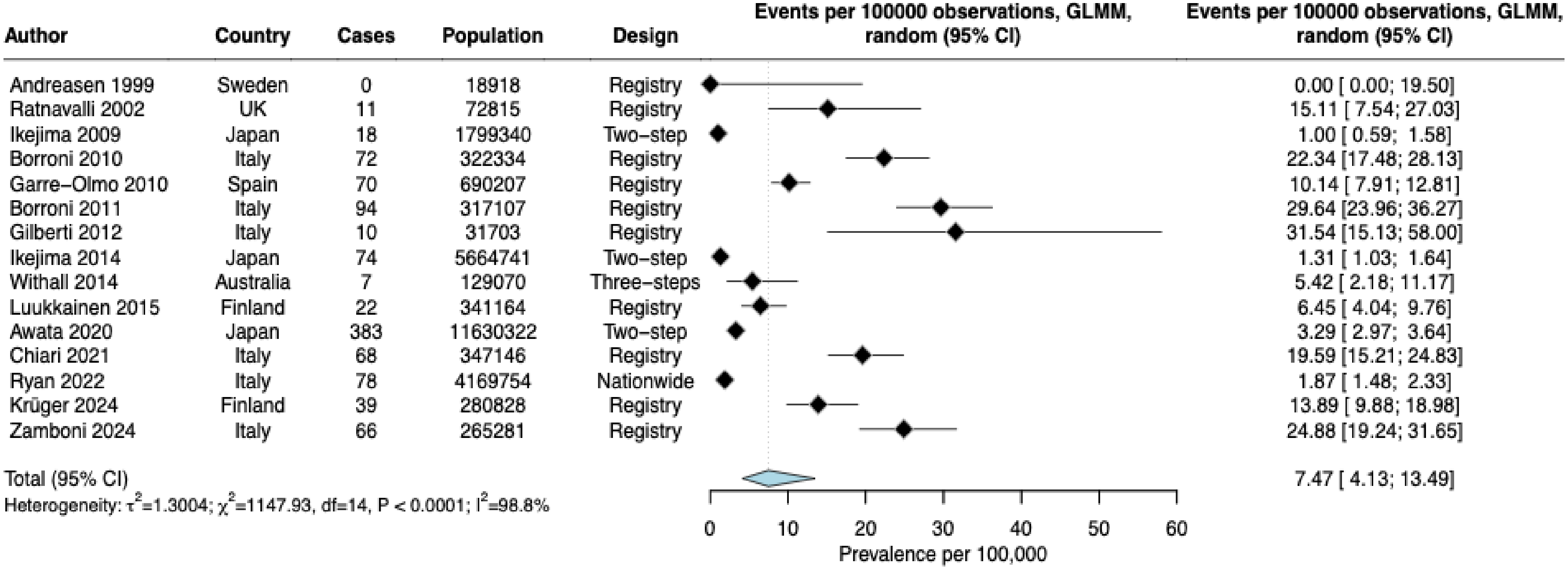

eFigure 7. Forest plot of studies incidence rates for >65 years.

Author	Country	Cases	Population	Events per 100000 observations, GLMM, (95% CI)
Gascón−Bayarri 2007	Spain	5	1754	285.06 [92.62; 663.98]
Gilberti 2012	Italy	32	21836	146.55 [100.26; 206.82]
Meguro 2012	Japan	1	14579	6.86 [0.17; 38.21]

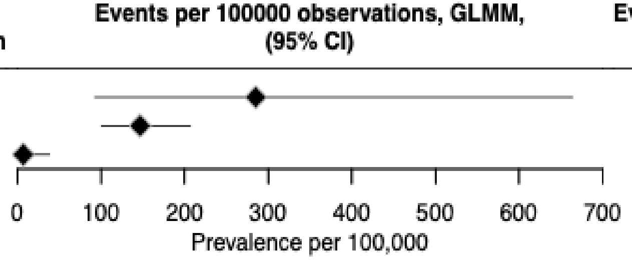

© 2025 Urso D et al. *JAMA Neurology*.

eFigure 8. Forest plot of studies prevalence rates for >65 years.

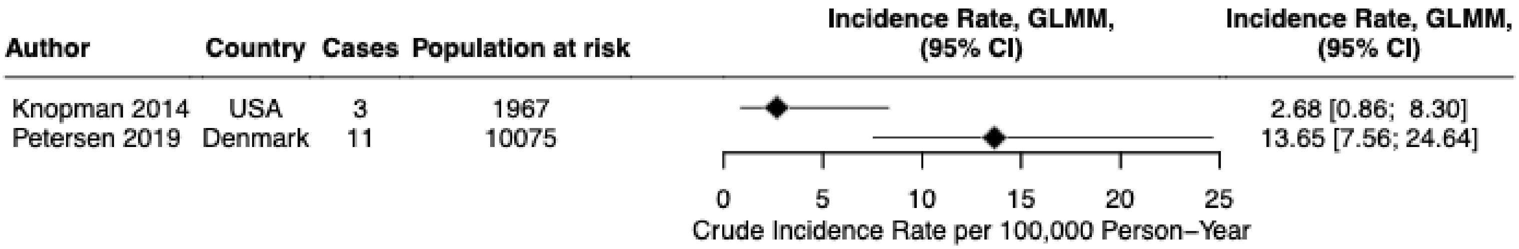

eFigure 9. Forest plot of sensitivity analysis: incidence estimates using population at risk defined by authors.

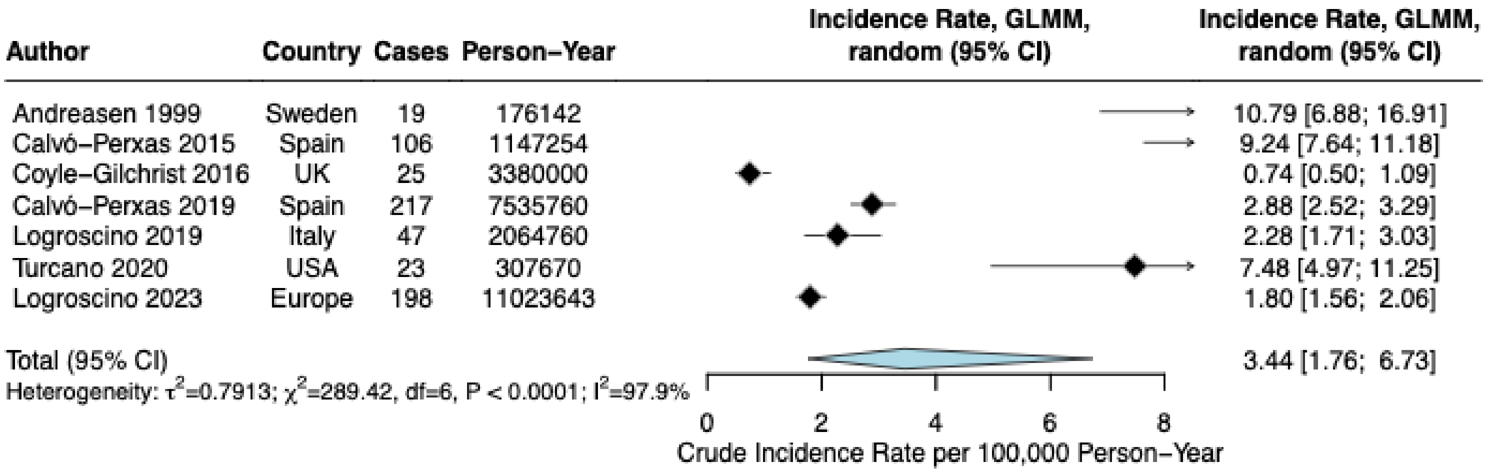

© 2025 Urso D et al. *JAMA Neurology*.

eFigure 10. Forest plot of sensitivity analysis: prevalence estimates using population at risk defined by authors.

Author	Country	Cases	Population	Events per 100000 observations, GLMM, random (95% CI)
Andreasen 1999	Sweden	8	29357	27.25 [11.77; 53.69]
Rosso 2003	Netherlands	55	2043949	2.69 [2.03; 3.50]
Borroni 2010	Italy	213	531249	40.09 [34.89; 45.85]
Gilberti 2012	Italy	42	119647	35.10 [25.30; 47.45]
Coyle–Gilchrist 2016	UK	95	1690000	5.62 [4.55; 6.87]
Total (95% CI)				13.82 [5.27; 36.26]

Heterogeneity: τ^2=1.1713; χ^2=483.71, df=4, P < 0.0001; I^2=99.2%

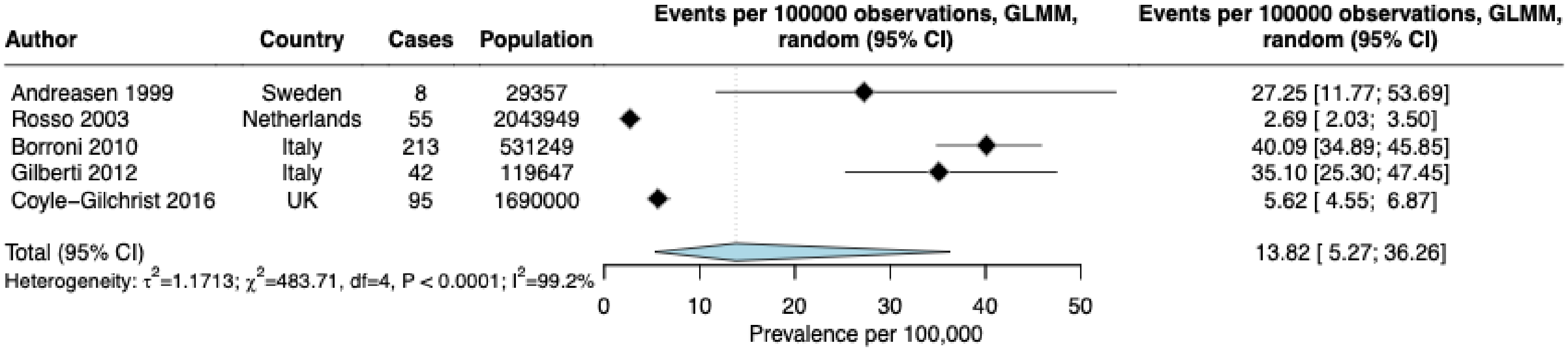

eFigure 11. Forest plot of cumulative incidence analysis.

Source	P–value	Tau2	Tau	I2	Rate (95% CI)
Adding Andreasen 1999 1999 (k=1)	.	.	.	.	5.12 [3.26; 8.02]
Adding Calvó–Perxas 2015 2015 (k=2)	.	0.0786	0.2803	87.1%	3.33 [2.06; 5.38]
Adding Coyle–Gilchrist 2016 2016 (k=3)	.	0.5832	0.7637	95.6%	2.09 [0.86; 5.09]
Adding Calvó–Perxas 2019 2019 (k=4)	.	0.4382	0.6620	94.1%	2.27 [1.16; 4.43]
Adding Logroscino 2019 2019 (k=5)	.	0.3378	0.5812	92.2%	2.27 [1.34; 3.84]
Adding Turcano 2020 2020 (k=6)	.	0.2898	0.5383	90.4%	2.38 [1.52; 3.74]
Adding Logroscino 2023 2023 (k=7)	.	0.2490	0.4990	91.6%	2.28 [1.55; 3.36]
Total	.	0.2490	0.4990	91.6%	2.28 [1.55; 3.36]

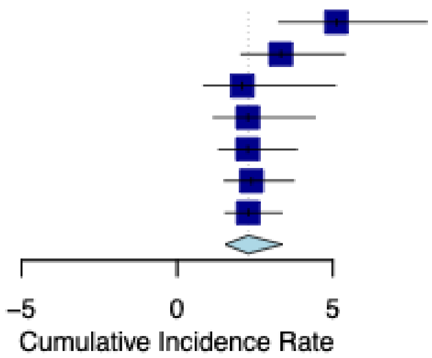

© 2025 Urso D et al. *JAMA Neurology*.

eFigure 12. Forest plot of cumulative prevalence analysis.

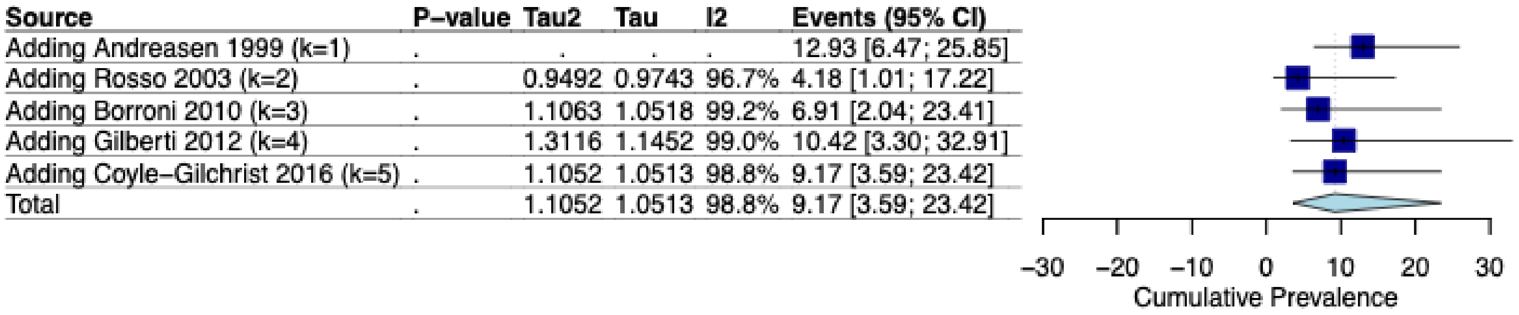

eFigure 13. Forest plot of leave-one-out analysis for incidence.

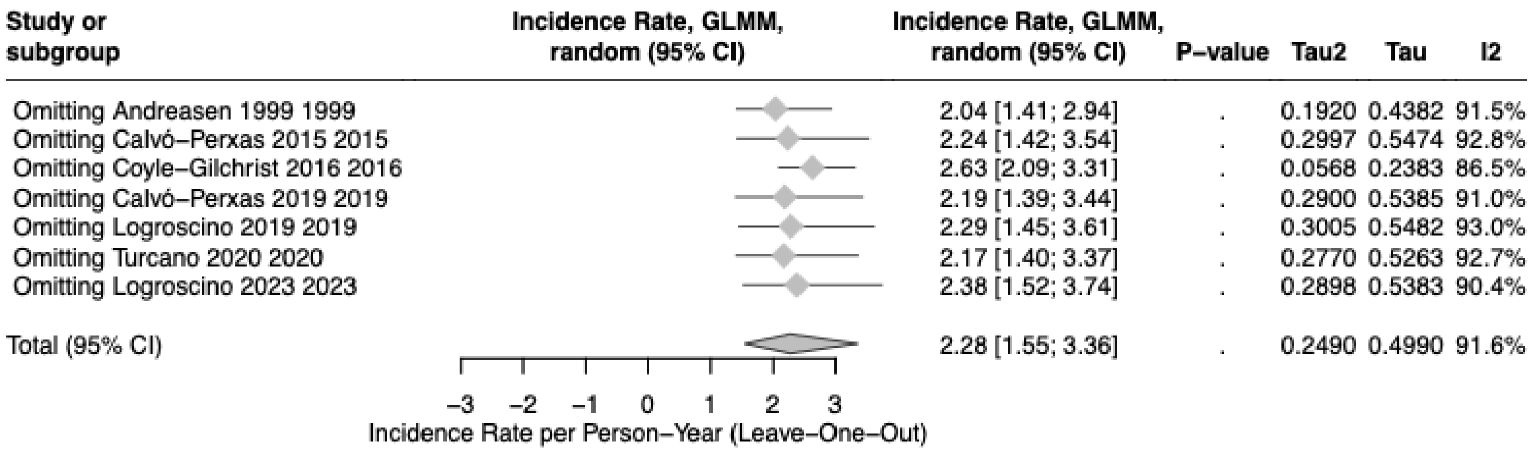

© 2025 Urso D et al. *JAMA Neurology*.

eFigure 14. Forest plot of leave-one-out analysis for prevalence

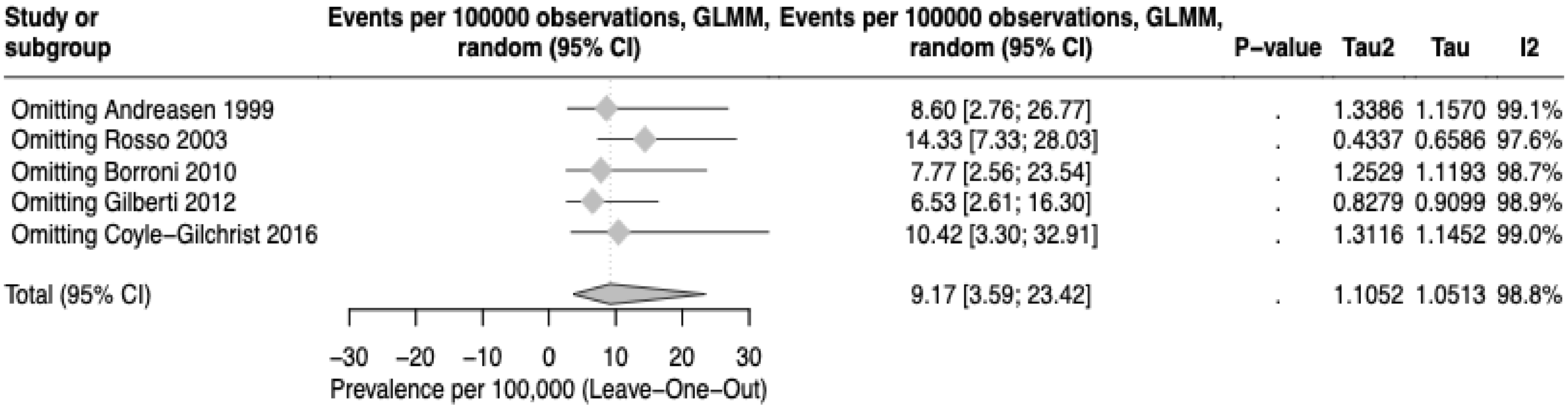

© 2025 Urso D et al. *JAMA Neurology*.
