# Supplementary material for: Incidence and Prevalence of Frontotemporal Dementia: A Systematic Review and Meta-Analysis
Source: JAMA Neurol. 2025 Sep 8;82(11):1144–52. doi: 10.1001/jamaneurol.2025.3307 (PMC12418226; doi:10.1001/jamaneurol.2025.3307)
Supplement: Supplement 2. — Data sharing statement [file jamaneurol-e253307-s002.pdf]

Data Sharing Statement

Urso. Incidence and Prevalence of Frontotemporal Dementia. *JAMA Neurol*. Published September 08, 2025. doi:10.1001/jamaneurol.2025.3307

Data
Data available: Yes
Data types: Deidentified participant data
How to access data: sgiannonil@gmail.com
When available: With publication

Supporting Documents
Document types: Statistical/analytic code
How to access documents: sgiannonil@gmail.com
When available: With publication

Additional Information
Who can access the data: anyone requesting the data
Types of analyses: for any purpose
Mechanisms of data availability: with investigator support
